# The importance of expert review to clarify ambiguous situations for (Q)SAR predictions under ICH M7

**DOI:** 10.1186/s41021-020-00166-y

**Published:** 2020-09-22

**Authors:** Robert S. Foster, Adrian Fowkes, Alex Cayley, Andrew Thresher, Anne-Laure D. Werner, Chris G. Barber, Grace Kocks, Rachael E. Tennant, Richard V. Williams, Steven Kane, Susanne A. Stalford

**Affiliations:** Lhasa Limited, Granary Wharf House, 2 Canal Wharf, Leeds, LS11 5PS UK

**Keywords:** (Q)SAR, ACEM, Ames, Derek Nexus, ICH M7, In silico, JEMS, Lhasa Limited, Mutagenicity, Sarah Nexus

## Abstract

The use of in silico predictions for the assessment of bacterial mutagenicity under the International Council for Harmonisation of Technical Requirements for Pharmaceuticals for Human Use (ICH) M7 guideline is recommended when two complementary (quantitative) structure-activity relationship (Q)SAR models are used. Using two systems may increase the sensitivity and accuracy of predictions but also increases the need to review predictions, particularly in situations where results disagree. During the 4th ICH M7/QSAR Workshop held during the Joint Meeting of the 6th Asian Congress on Environmental Mutagens (ACEM) and the 48th Annual Meeting of the Japanese Environmental Mutagen Society (JEMS) 2019, speakers demonstrated their approaches to expert review using 20 compounds provided ahead of the workshop that were expected to yield ambiguous (Q)SAR results. Dr. Chris Barber presented a selection of the reviews carried out using Derek Nexus and Sarah Nexus provided by Lhasa Limited. On review of these compounds, common situations were recognised and are discussed in this paper along with standardised arguments that may be used for such scenarios in future.

## Introduction

The International Council for Harmonisation of Technical Requirements for Pharmaceuticals for Human Use (ICH) M7 guideline provides guidance on the assessment and control of DNA reactive (mutagenic) impurities in pharmaceuticals [1]. In the absence of experimental data, ICH M7 recommends in silico assessment of bacterial mutagenicity through use of two complementary (quantitative) structure-activity relationship ((Q)SAR) methodologies, namely expert rule-based and statistical. The guideline further states that predicted results may warrant review with “…*the use of expert knowledge in order to provide additional supportive evidence on relevance of any positive, negative, conflicting or inconclusive prediction and provide a rationale to support the final conclusion*”. In this manner, expert review of the predictions can improve the predictive performance of (Q)SAR [2] to give a level of accuracy which compares favourably to the reproducibility of the Ames test [3, 4]. In the absence of a specified procedure within ICH M7 [1], frameworks for carrying out expert reviews have subsequently been proposed [5–7].

The 1st ICH M7/QSAR Workshop was held by the Japanese Environmental Mutagen Society (JEMS) and the Bacterial Mutagenicity Study Group (BMS) in 2016 to understand the use of (Q)SAR tools, and expert review, by industry for regulatory purposes under ICH M7 [8]. Since then, the ICH M7/QSAR Workshop, including case studies of expert review, has been held annually by JEMS. The 4th ICH M7/QSAR Workshop was held during Asian Congress on Environmental Mutagens (ACEM)/JEMS 2019, a workshop dedicated to discussion of ICH M7 [9]. The workshop focused on two parts of the guideline. Part 1 discussed “*QSAR prediction and expert judgement for Ames mutagenicity*” while part 2 discussed “*control of impurities in pharmaceuticals by purge factor*”. Prior to the workshop participants were asked to provide predictions and expert review for 20 compounds expected to yield ambiguous (Q)SAR results [9]. Speakers during part 1 were then able to demonstrate any interesting approaches they used to undertake expert review of these predictions.

### QSAR prediction and expert judgement for Ames mutagenicity session

**Fig. 1:**
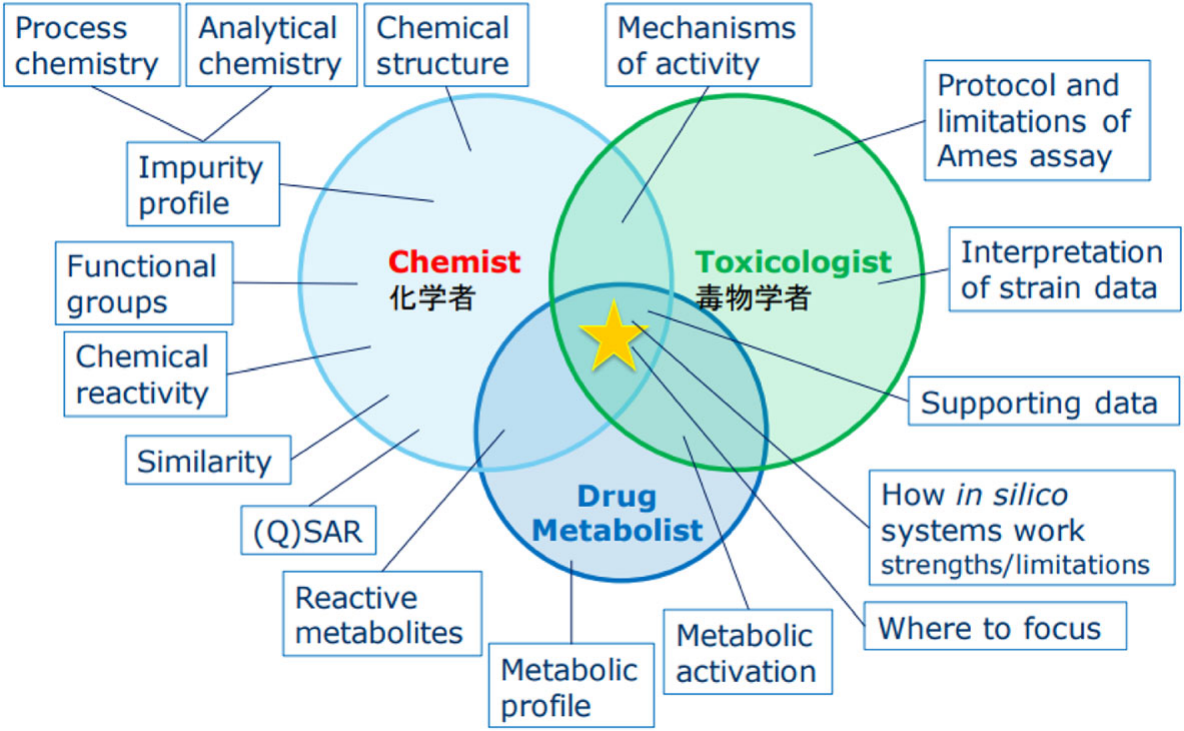
Skills required by scientist(s) to undertake expert review as presented by Dr. Chris Baber at the 4th ICH M7/QSAR Workshop

In silico predictions should be considered as just one of the pieces of evidence used to support an overall conclusion of mutagenic activity (or lack thereof), and assessing confidence in the predictions requires specialist knowledge about the query structure and analogous compounds. Understanding the chemical structure enables the user to consider the expected reactivity of the compound and therefore select relevant chemicals for comparison that have the same functional group(s) and reactivity. Understanding of the protocol, interpretation and limitations of the Ames test enables read-across of Ames data for these selected compounds. Understanding of drug metabolism allows a reasoned assessment of the likeliness of the drug to undergo metabolic (de)activation that adjusts expected mutagenic potential. Therefore, it is important that the scientist(s) implementing the expert review has expertise in genetic toxicology as well as complementary disciplines such as chemistry and drug metabolism (Fig. 1).

Well-designed (Q)SAR systems should present the accompanying reasoning for predictions in a transparent and accessible manner to enable review. Expert rule-based systems should have alerts that emulate the human decision-making process, benefiting from related and/or tacit knowledge about a chemical class which can include knowledge of proprietary data. The output may be enriched with details of known (non)-mutagens to elicit mechanistic descriptions and reasons for exclusions to the structural alert. Statistical-based systems should be driven by the data set without interference of the human expert, benefitting from the capability to use large datasets to identify trends beyond the analysis of a human expert. Understanding the benefits of each system [10] is essential to undertake an effective review to make an assessment of a query compound that is reasoned and consistent with knowledge and data for relevant analogues.

The level of expert review required will depend on the obtained predictions [5]. When predictions agree, they are likely to be relatively simple to review. Elsewise, should predictions be conflicting, inconclusive or absent, expert review is more difficult. In these situations, it is often still possible to find additional evidence to resolve into an overall call; however, sometimes the review is still inconclusive and it is advisable to test the substance or rely on a purge argument under ICH M7 option 4 which allows a calculation to demonstrate the level of the impurity in the drug substance will be below the acceptable intake and does not require analytic testing. Lhasa Limited has developed software which can help in these scenarios such as Vitic, a structure searchable database containing high-quality toxicity data, and Mirabilis [11], a risk assessment tool incorporating an industry-standardised approach for calculating purge factors.

Lhasa provided predictions and expert review by processing the provided compounds using the ICH M7 functionality in Nexus v.2.2.2, which gives predictions from the expert rule-based system Derek Nexus v.6.0.1 (certified knowledge base 2018 1.1) and the statistical-based Sarah Nexus v.3.0.0 (certified model 2.0). Predictions in Derek Nexus were considered where endpoint = mutagenicity in vitro and species = bacterium, and any compound activating an alert with a reasoning level of “equivocal” or above was treated as a positive prediction. Default prediction settings were used for Sarah Nexus.

**Fig. 2:**
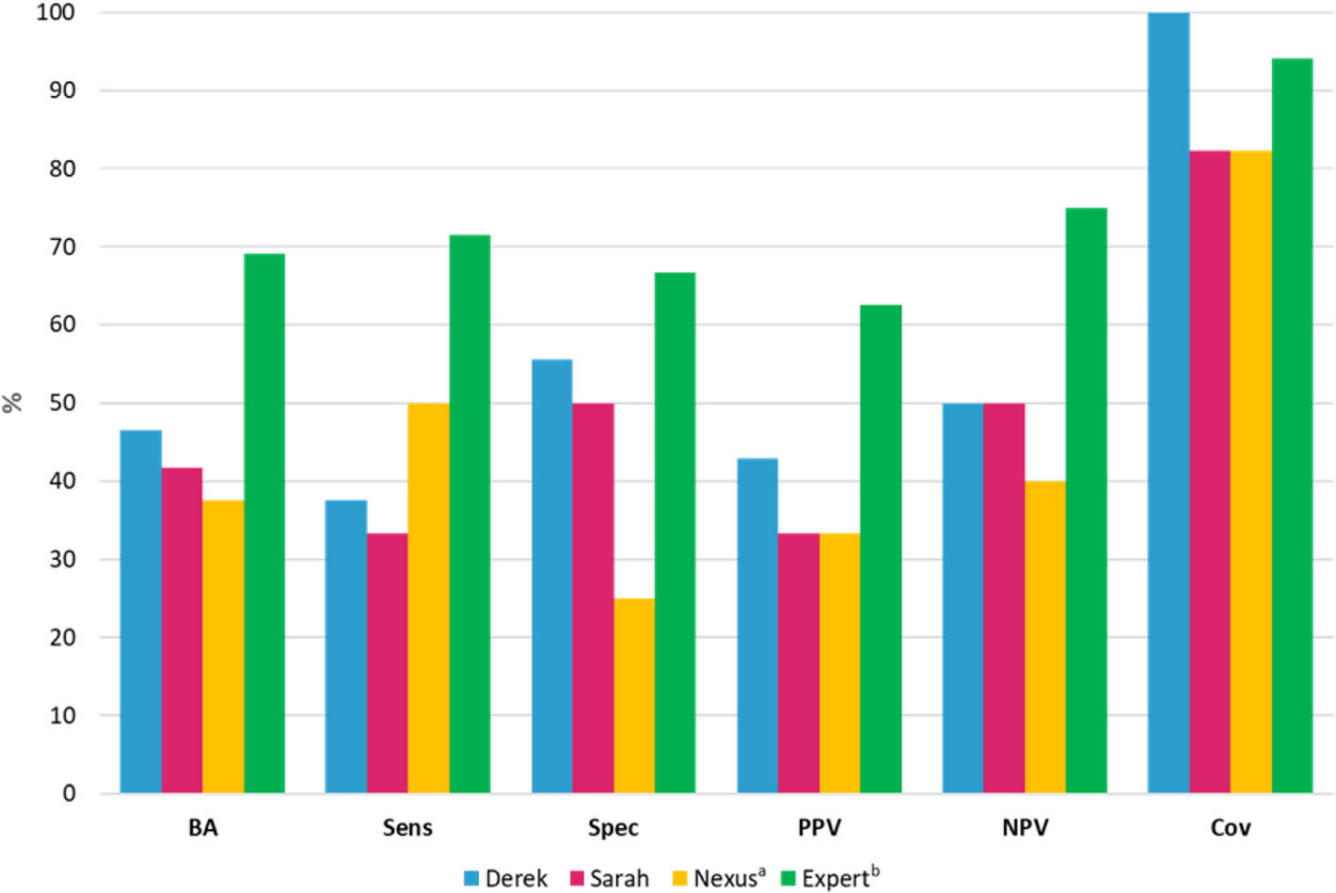
Performance statistics for (Q)SAR predictions, before and following expert review, against compounds provided ahead of the workshop expected to be yield ambiguous (Q)SAR predictions. BA = balanced accuracy ($\frac{Sens+ Spec}{2}$), Sens = sensitivity ($\frac{TP}{TP+ FN}$), Spec = specificity ($\frac{TN}{TN+ FP}$), PPV = positive predictive value ($\frac{TP}{TP+ FP}$), NPV = negative predictive value ($\frac{TN}{TN+ FN}$), Cov = coverage ($\frac{TP+ FP+ TN+ FN}{TP+ FP+ TN+ FN+ Eq+ OD}$). ^a^Conservative call made by combination of predictions from Derek Nexus and Sarah Nexus. ^b^Expert call following review of predictions from Derek Nexus and Sarah Nexus

Table 1 and Fig. 2 highlight the overall performance of the two systems, before and after expert review, against the experimental data for 17 of the 20 compounds. Three compounds containing a carboxylic acid halide were removed from the analysis as the Ames test may not be applicable for these compounds as described by Amberg et al. [12] and discussed later. The results from Derek Nexus and Sarah Nexus were combined using a conservative approach (i.e. a positive call from either system was called positive) to yield the Nexus result. The expert result is the call assigned following expert review of the predictions, using information presented in the Derek Nexus and Sarah Nexus predictions as well as publicly available Ames data for similar compounds and knowledge of chemical reactivity where appropriate. The use of “equivocal” has various meanings depending on the model being used. An equivocal result is provided by Sarah Nexus when the “*confidence level is below which a prediction of positive or negative is unable to be made*”. Using a conservative approach, the Nexus equivocal result is assigned when Derek Nexus provides a negative prediction and Sarah Nexus provides an equivocal prediction. The expert call uses equivocal when the prediction is unable to be resolved to either positive or negative.

**Table 1:** Performance statistics for (Q)SAR predictions, before and following expert review

Model	TP	FP	TN	FN	Eq	OD
Derek Nexus	3	4	5	5	0	0
Sarah Nexus	2	4	4	4	1	2
Nexus^a^	3	6	2	3	1	2
Expert^b^	5	3	6	2	1	0

*TP* True positive, *FP* False positive, *TN* True negative, *FN* False negative, *EQ* Equivocal, *OD* Outside domain.

^a^Conservative call made by combination of predictions from Derek Nexus and Sarah Nexus

^b^Expert call following review of predictions from Derek Nexus and Sarah Nexus

The results are a notable example of how expert knowledge can add value to in silico predictions when considering reduction in both the number of false positives and false negatives. However, the increases in sensitivity and specificity should be treated with caution considering the degree of change expected for a small sample size. It is also important to note that the predicted results have lower accuracy than is usually observed for in silico prediction of Ames mutagenicity as this is a small sample of compounds selected as highly challenging for (Q)SAR systems to predict accurately and are expected to yield ambiguous (Q)SAR predictions.

This exercise was an interesting opportunity to highlight some specific scenarios and how an expert using software by Lhasa may resolve the 2 (Q)SAR predictions. Although those highlighted below show disagreement between the two systems, this is not the common scenario. Analysis of Derek Nexus and Sarah Nexus against a number of datasets showed concordance in 70–85% of predictions with accuracy as high as 90% for concordant results [5].

### Case studies

**Fig. 3:**
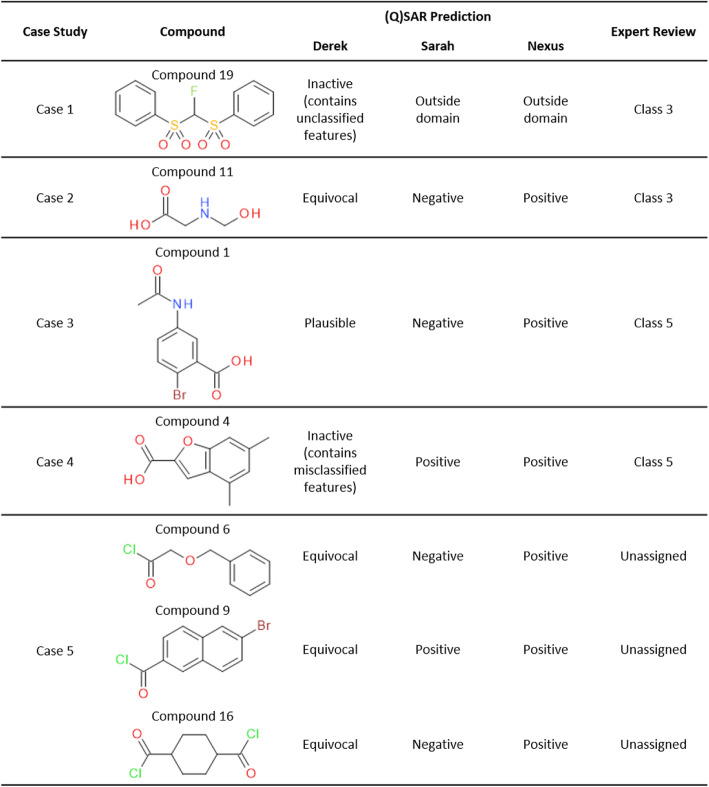
Compounds used as case studies to illustrate common prediction scenarios. (Q)SAR predictions show the predictions provided by Derek Nexus, Sarah Nexus and a conservative call considering both prediction results. Expert review shows the ICH M7 class assigned following review of the (Q)SAR predictions by the authors

Figure 3 highlights the predictions generated for certain compounds that illustrate common prediction scenarios that will be discussed as case studies in the following sections. (Q)SAR predictions show the results from Derek Nexus and Sarah Nexus as well as a conservative call which considers both (Q)SAR predictions. The expert review shows the ICH M7 class assigned based on review of the predictions by the authors of this paper. Under the guidelines, Class 3 impurities are defined as “*Alerting structure, unrelated to the structure of the drug* substance” whereas Class 5 are defined as “*No structural alerts, or alerting structure with sufficient data to demonstrate lack of mutagenicity or carcinogenicity*” [1]. A discussion detailing why the use of (Q)SAR predictions may not be appropriate for carboxylic acid halides will be presented to demonstrate why no ICH M7 classifications were assigned for case 5.

#### Case 1: unclassified feature identified by expert rule-based system cannot be adequately assessed

##### Overall (Q)SAR prediction: outside domain

Derek Nexus provides a negative prediction for compound 19 but highlights that it contains an unclassified feature. When a compound does not activate an alert for mutagenicity in Derek Nexus, the compound is then compared to a reference set of compounds with known Ames results to conclude if the compound contains (1) a feature shared exclusively with known false negatives or (2) a feature that is novel or unknown to the model [13]. In this case, the unclassified feature means that there are no compounds in the reference library that also contain the highlighted feature (*N*-fluoro-*N*-bis-sulfonamide), reducing confidence in the negative prediction.

Sarah Nexus provides a prediction of outside domain, also highlighting that the *N*-fluoro-*N*-bis-sulfonamide is not present in compounds in the training set.

##### Expert review ICH M7 classification: class 3

No additional information was found relating to mutagenic activity of *N*-fluoro-*N*-bis-sulfonamides to provide support for or against the Derek Nexus prediction. In the absence of relevant supporting examples, an assessment of the expected chemical reactivity is considered as it is an important factor in the direct reaction with DNA to form adducts and produce mutations. In this respect, compound 19 is an electrophilic fluorinating reagent and expert review concluded this should be assigned class 3 as the feature for which there is a lack of Ames data may prove to be a potential toxicophore.

### Experimental result: positive

#### Case 2: Toxicophore identified by expert rule-based system is not adequately assessed by statistical system

##### Overall (Q)SAR prediction: positive

Derek Nexus provides a prediction of equivocal for compound 11; equivocal has a specific meaning in Derek Nexus in that there is evidence both for and against activity [14], when making conservative predictions it is recommended to consider this a positive prediction with limitations that requires review. The class of compounds, *N*-methylols, are reported to be mutagenic with the most likely mechanism requiring hydrolysis to formaldehyde which subsequently reacts with DNA [15]. The alert description provides positive and negative examples, and notes that the Ames test may be inadequate for this class of compounds due to cytotoxicity and/or low rate of conversion to formaldehyde which is itself not a potent mutagen [16–18].

Sarah Nexus provides a prediction of negative with good confidence (58%). The confidence metric in Sarah is presented on a scale from 0 to 100% and positively correlates with accuracy, hence 58% confidence is expected to be approximately 75% accurate [19]. The prediction is supported by negative hypotheses for carboxyl and alkoxyl functional groups but not *N*-methylol. Furthermore, no *N*-methylol-containing compounds are in the training set examples.

### Expert review ICH M7 classification: class 3

Considering the negative prediction provided by Sarah Nexus is not supported by examples containing the *N*-methylol functional group, it is not assessing the toxicophore identified by Derek Nexus. Derek Nexus gives a clear, mechanistically detailed rationale for activity which makes for an easy expert assessment to dismiss the statistical prediction and conclude the compound should be classified as class 3.

### Experimental result: positive

#### Case 3: Toxicophore identified by expert system can be adequately negated by most similar compounds in statistical system

##### Overall (Q)SAR prediction: positive

Derek Nexus provides a positive prediction for compound 1 as it activates the alert “Aromatic amine or amide”. On reading of the accompanying alert comments, it is noted that the mechanism of action of amides in the Ames test is attributed to the amine metabolite which itself undergoes *N*-hydroxylation followed by *O*-esterification to give rise to a nitrenium ion that is the ultimate mutagenic species [20]. Furthermore, the alert comments state “*…anilines substituted with strong or moderate electron-withdrawing groups…are not mutagenic in the Ames test.*”. Therefore, although compound 1 does not match this exclusion, the presence of the bromine *para* to the amide warrants investigation as an electron withdrawing group that may reduce activity.

**Fig. 4:**
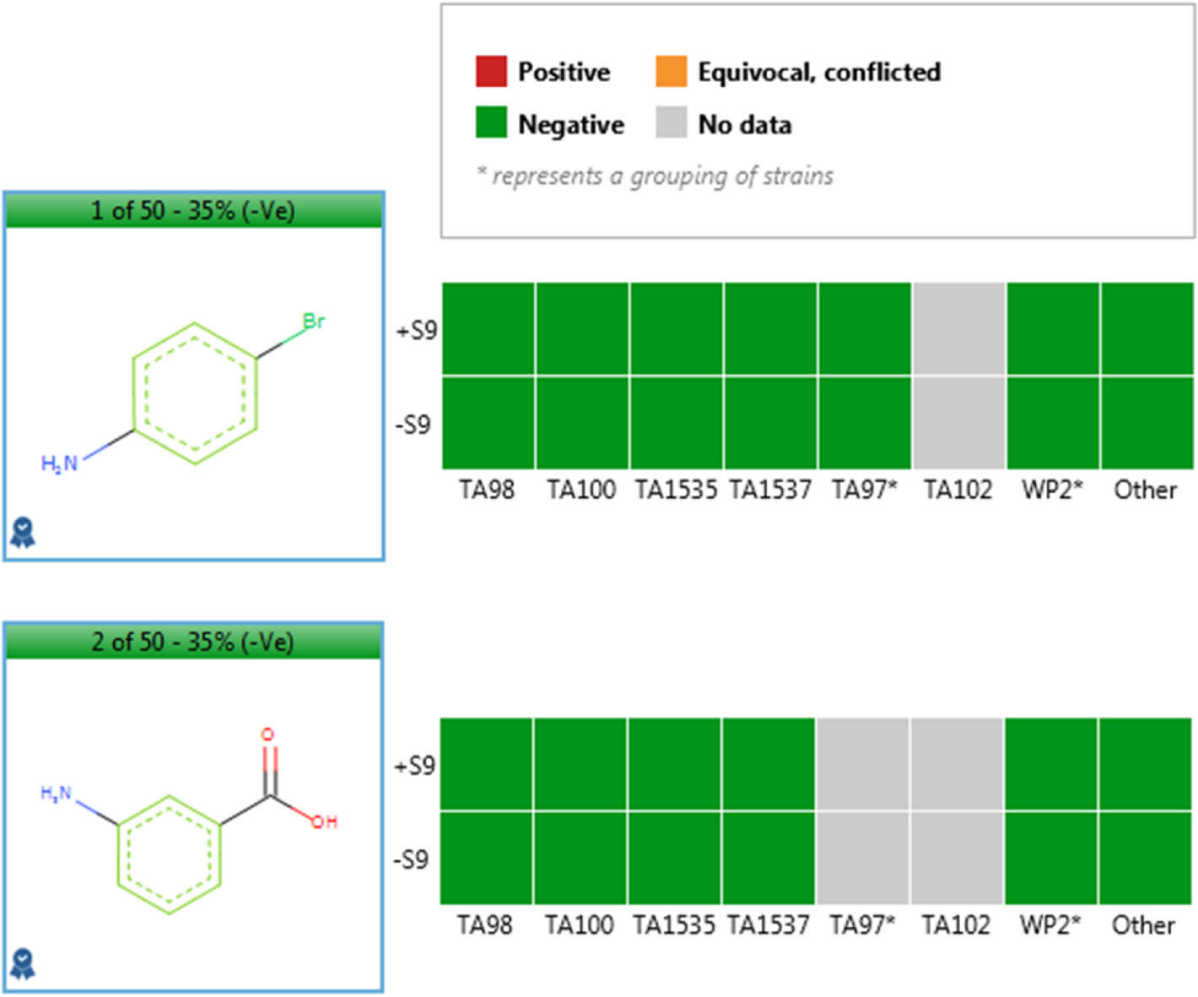
Experimental Ames test strain profile data presented in Sarah Nexus for 4-bromoaniline and 3-aminobenzoic acid

Sarah Nexus provides a negative prediction with a confidence score of 40% which is expected to be approximately 70% accurate [19]. Sarah Nexus also provides a negative prediction with 31% confidence for the expected amine metabolite, 2-bromo-5-aminobenzoic acid. Moreover, inspection of the training set examples identifies 4-bromoaniline and 3-aminobenzoic acid as non-mutagens, each having been tested in multiple strains (Fig. 4).

### Expert review ICH M7 classification: class 5

The comments accompanying the Derek Nexus prediction suggest compound 1 narrowly misses an exclusion and also highlights the borderline mutagenic activity of multiply substituted aromatic amines and amides, making them a significant challenge to in silico tools [21]. Derek Nexus described that activity is expected to result from metabolic activation, initially to the amine. In this situation, training set examples in Sarah Nexus provide evidence that the expected aromatic amine metabolite is likely to be non-mutagenic as 4-bromoaniline and 3-aminobenzoic acid are both known non-mutagens. Therefore, it is possible to use the relevant analogues to support overturning the Derek Nexus prediction to assign the ICH M7 classification as class 5.

### Experimental result: negative

#### Case 4: Toxicophore identified by statistical system is not causative of activity of supporting training set examples

##### Overall (Q)SAR prediction: positive

**Fig. 5:**
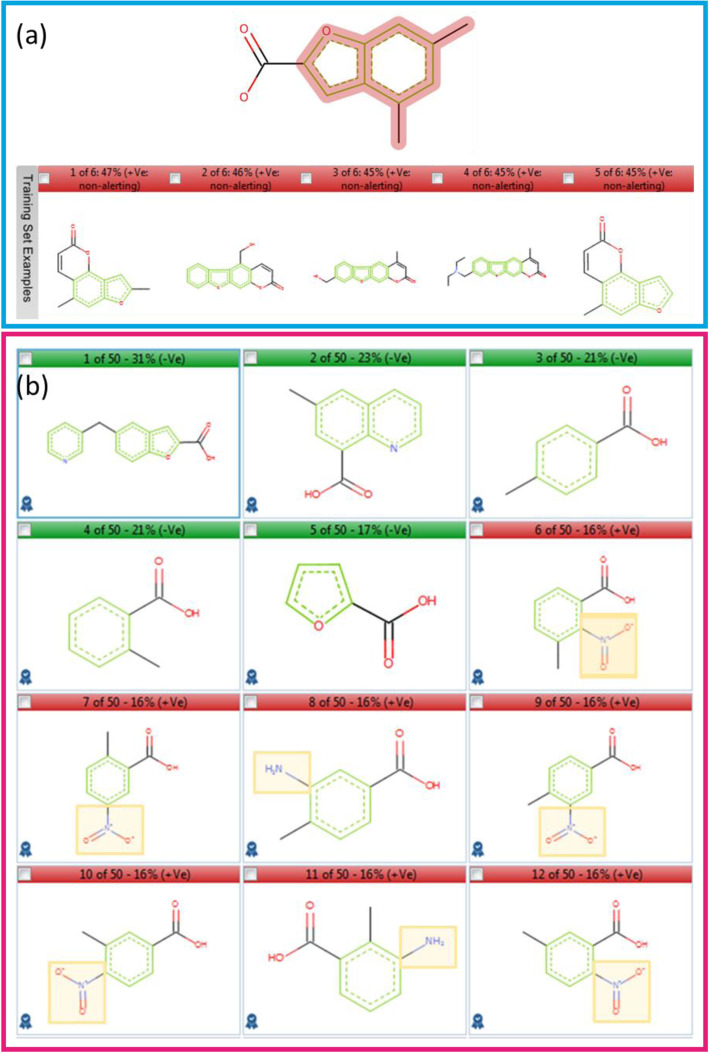
Example compounds displayed for predictions of compound 4 in Nexus; **a** highlighted misclassified feature and associated reference compounds in Derek Nexus; **b** most similar training set examples in Sarah Nexus highlighting functional groups causative of activity yet not present in the query. Example compounds in red boxes and green boxes are positive and negative in the Ames test respectively. Functional groups highlighted in yellow boxes are alerting features absent from the query compound

Derek Nexus provides a negative prediction for compound 4 but highlights that it contains a misclassified feature. A misclassified feature is one contained in compounds in the Lhasa Ames Test Reference Set that are active in the Ames test, reducing confidence in the negative prediction [13]. Figure 5 shows the highlighted feature (in red) and a selection of the compounds identified in the reference set. In this case, those analogues are all furocoumarins that activate an alternative alert in Derek Nexus that contains an accompanying description of a specific photomutagenicity mechanism [22] that is not relevant to the query compound, since it does not have an extended aromatic ring system necessary to provide a chromophore.

Sarah Nexus provides a positive prediction with low confidence (7%). The obtained hypothesis assesses only the carboxylic acid functional group and is normally associated with a negative signal in the training set. However, the examples most relevant to the query are a mixture of positive (red boxes) and negative (green boxes) that overrule the negative signal (Fig. 5). It is notable that the most similar compound contains the benzofuran-2-carboxylic acid function in compound 4 and is non-mutagenic, whereas close inspection of the mutagens shows their activity is associated with functional groups absent from the query that would activate other alerts in Derek Nexus i.e. aromatic amine and nitro highlighted in yellow boxes.

### Expert review ICH M7 classification: class 5

The inactive prediction provided by Derek Nexus should be accepted because the mutagenic activity of furocoumarins in the Lhasa Ames Test Reference Set is associated with an alternative toxicophore. Although Sarah Nexus provides a positive prediction, the activity of the mutagenic examples is associated with toxicophores not in compound 4. If these examples are removed from the training set then those remaining would support a negative prediction, including the most similar compound, furegrelate sodium (Fig. 5b, Example 1), which is negative when tested in 5 strains [23]. Therefore, it is possible to use the relevant analogues to support overturning the Sarah Nexus prediction to assign the ICH M7 classification as class 5.

### Experimental result: negative

#### Case 5: Ames test cannot adequately assess the hazard of the query compound

##### Overall (Q)SAR prediction: positive

Derek Nexus provides a prediction of equivocal for compounds 6, 9 and 16, which should be treated as a positive prediction with limitations that require review. Although the ICH M7 guideline uses the Ames test to assess mutagenic potential, the alert comments describe the ambiguity of results for carboxylic acid halides which provide non-reproducible positive results [12]. Positive activity for some carboxylic acid halides in the Ames test is thought to depend on the solvent used. Carboxylic acid halides are known to react with DMSO and water when either is used as the vehicle. Reaction with DMSO yields halodimethyl sulfides (HDMS), reactive alkylating agents known to be responsible for activity, whereas reaction with water causes hydrolytic decomposition and inactivity. Non-DMSO organic solvents are generally thought to be most appropriate, yielding negative results for several compounds observed as active when tested in the presence of DMSO. To reflect the dependence of activity on the test vehicle, the reasoning level in Derek Nexus is set to equivocal.

For none of compounds 6, 9 or 16 was the carboxylic acid halide group considered as a hypothesis by Sarah Nexus. Compound 9 is predicted positive with low confidence (2%) by Sarah Nexus and analysis of the training set examples shows positive activity is associated with other toxicophores. Conversely, compounds 6 and 16 are predicted negative with moderate confidence, 26 and 17% respectively. Compound 6 is supported by two training set examples that are carboxylic acid chlorides, phenylacetyl chloride and 3-phenylpropionyl chloride, which have been assigned overall negative calls based on negative Ames data from tests in acetonitrile and positive results in DMSO [12]. Compound 16 is not supported by training set examples that are carboxylic acid halides; however, two similar compounds were discovered in the public literature [12]. In line with the previous discussion, 1-(2-ethylbutyl)cyclohexanecarbonyl chloride returned a positive result when in DMSO whereas 1-methyl-1-cyclohexanecarboxylic acid chloride returned a negative result tested in ethylene glycol dimethyl ether (DME).

### Expert review ICH M7 classification: unassigned

In the case of carboxylic acid halides, a significant degree of review is required including analysis of the specific protocol considering the variability of results in different solvents [12]. However, it is important to consider that other functional groups may still contribute to mutagenic activity and require review. It is also worth noting that the chemical reactivity of carboxylic acid halides means they are likely to be purged at the stage of their introduction during synthesis and in subsequent handling steps which means they could be controlled under ICH M7 option 4 [1]. This may present a more practical method for controlling carboxylic acid halides rather than assigning an ICH M7 classification based on (Q)SAR predictions as, in the absence of other alerting features, it is possible that experts may interpret their activity differently.

### Experimental result: compound 6: negative (acetone), compound 9: positive (DMSO), compound 16: negative (1,4-dioxane)

#### Summary

In the absence of experimental data, ICH M7 recommends in silico assessment of bacterial mutagenicity through use of two complementary (Q)SAR methodologies [1]. Prediction review using expert knowledge is a process known to improve the predictive performance [2]. Published frameworks to undertake such reviews share common principles and arguments; however, the level of expert review required will vary depending on the combination of prediction results and level of supporting data presented by the models and associated databases [5–7]. Furthermore, the classification concluded by different experts may vary depending on their judgement of the available data.

During the 4th ICH M7/QSAR Workshop held during ACEM/JEMS 2019, speakers demonstrated their approaches to expert review of 20 compounds selected to be challenging based upon ambiguous (Q)SAR results [9]. A subset of the 20 predictions were selected to demonstrate common scenarios occurring when 2 complementary (Q)SAR models are used and standardised arguments for their resolution to a single prediction. The expert should review each prediction independently and in respect to each other considering factors such as the confidence/likelihood presented alongside the result, similarity of example compounds to the query, overlap of the alerting feature in both systems, and knowledge of chemical reactivity, biological activity and applicability of the Ames test for the chemical class. The available knowledge from each system will ultimately determine whether a prediction from an expert rule-based or statistical model is overruled. However, for some chemicals classes, such as carboxylic acid halides, it may be more relevant to present an argument to control such impurities under ICH M7 option 4.

## Data Availability

Not applicable.

## Abbreviations

(Q)SAR: (Quantitative) structure-activity relationship
ACEM: Asian Conference on Environmental Mutagens
BA: Balanced accuracy
BMS: Bacterial Mutagenicity Study Group
Cov: Coverage
DME: Ethylene glycol dimethyl ether
DMSO: Dimethylsulfoxide
FN: False negative
FP: False positive
HDMS: Halodimethyl sulfides
ICH: International Council for Harmonization of Technical Requirements for Pharmaceuticals for Human Use
JEMS: Japanese Environmental Mutagen Society
NPV: Negative predictive value
OD: Outside domain
PPV: Positive predictive value
Sens: Sensitivity
Spec: Specificity
TN: True negative
TP: True positive
